# Skin Marks in Critically Endangered Taiwanese Humpback Dolphins (*Sousa chinensis taiwanensis*)

**DOI:** 10.3390/ani13040608

**Published:** 2023-02-09

**Authors:** Yun Ho, Pei-Ying Wu, Lien-Siang Chou, Wei-Cheng Yang

**Affiliations:** 1 Institute of Ecology and Evolutionary Biology, National Taiwan University, Taipei 106, Taiwan; 2 Department of Veterinary Medicine, School of Veterinary Medicine, National Taiwan University, Taipei 106, Taiwan

**Keywords:** Taiwanese humpback dolphin, *Sousa chinensis taiwanensis*, skin mark, endangered

## Abstract

**Simple Summary:**

The population of Indo-Pacific humpback dolphin (*Sousa chinensis*) endemic to the inshore and estuarine waters of western Taiwan was identified as a subspecies (*Sousa chinensis* ssp. *taiwanensis*) and designated as “Critically Endangered” by the International Union for Conservation of Nature (IUCN). This population is at a high risk of extirpation, faces being impacted by some major anthropogenic threats, and numbers fewer than 65 individuals. Through a combination of long-term observations and photographic monitoring of identifiable individuals and stranding responses, accurate identification and classification of human-induced injuries and skin lesions is an effective approach and is particularly important to the management of the declining population. The results showed that the prevalence of skin marks is higher or comparable to the previous studies on the coastal cetacean populations across the globe. As the total population comprises dozens of individuals, radical measures to conserve the population and reduce environmental hazards are considered indispensable.

**Abstract:**

As long-lived apex predators, Indo-Pacific humpback dolphins (*Sousa chinensis*) are key indicators of marine coastal environmental health. Skin marks can be observed on dorsal body surfaces that are visible during mark–recapture studies that rely on photo-identification (photo-ID) methods. Skin mark prevalence may be an indicator of environmental or anthropogenic stressors in the ecosystem, which may lead to individual and/or population-level health concerns. The prevalence of marks of anthropogenic origin was assessed in the Critically Endangered *S. chinensis taiwanensis* population along the coasts of central Taiwan. Fifty, twenty-eight, and thirty-four individuals were identified in 2018, 2019, and 2021, respectively. At least one category of injuries was observed in 47 of 57 distinctive individuals (82%), and adults showed a higher prevalence of deep injuries than the other coloration stages. At least one category of skin lesion was observed in 33 of 57 distinctive individuals (58%), and high prevalence of skin lesions was found in mature individuals. Given the difficulty in taking direct observations, skin mark prevalence is proposed as a proxy for estimating habitat health and anthropogenic stressors upon *S. chinensis taiwanensis*. The moderate-to-high prevalence of skin marks in this study was designated as a warning of risks. This study provides important updated information for the assessment of the health and survival of this population. More effective management measures are urgently needed to reverse the observed population decline.

## 1. Introduction

Indo-Pacific humpback dolphins (*Sousa chinensis*) dwell in tropical and subtropical close shore waters belonging to the Indian Ocean and Pacific Oceans from northern Australia, the southern parts of China, extending to southeastern Asia, and around the borders of the Indian Ocean, extending to southern Africa [1]. *S. chinensis* is red-listed by the International Union for Conservation of Nature (IUCN) as ‘Near Threatened’, predominantly due to heavy fishing pressure, which results in incidental mortality and the loss of living space alongshore and in estuarine parts [2]. The population of *S. chinensis* was first reported along the coastal regions of western Taiwan, predominantly during 2004 [1]. The line-transect data from 2002 to 2004 estimated the population size may be around 99 (CV = 51.6%; 95% CI = 37 to 266) [3]. Being a small, diminishing, and geographically isolated population that faces numerous threats, this population was identified as a subspecies (*Sousa chinensis* ssp. *taiwanensis*) and designated as “Critically Endangered” by the Red List of Threatened Species of the IUCN [4]. This population is at a high risk of extirpation and faces being impacted by some major anthropogenic threats, including fisheries bycatch, chemical and biological pollution, noise, habitat loss, and degradation (reviewed in [5]).

To better understand marine ecosystem health and the potential impacts of environmental stressors, a range of species has been selected based on their life history traits to serve as sentinels [6]. As long-lived, apex predators, *S. chinensis* are key indicators of marine coastal environmental health. Long-term surveillance of local dolphins can potentially offer more information regarding the water quality and pollutants in the water, as small dolphins bioaccumulate pollutants from their prey in their blubber [7]. While there are many logistical challenges to assessing dolphin health in the wild, the presence of skin marks or lesions provides a visible morphological indicator of potential underlying disease [8]. Dermal marks are seen on the dorsal aspect of the body, which is observed during a mark–recapture analysis that counts on photo-identification (photo-ID) methods, as it is the most common noninvasive monitoring method to examine, identify, monitor, and classify skin changes in a free-ranging dolphin population. This provides an effective means to audit and compare minimum prevalence estimates and to track mark progression over time [9]. Cetacean skin marks were documented in many species of cetaceans around the globe [9] and revealed a broad spectrum of causes. Lesions caused by infectious diseases as well as environmental factors, injuries inflicted by sharks or any parasitic copepods/diatoms, post-traumatic scars and injuries resulting from propellers or fishing gear, and biological and chemical pollutants may contribute to skin mark development in cetaceans (reviewed in [10]). Lesions may be viral, fungal, or bacterial in origin, and their prevalence and severity can be influenced by environmental factors, such as sea surface temperature and salinity, and anthropogenic influences, including chemical pollutants [11,12,13,14,15,16]. While entanglements in fishing gear often result in fatalities for dolphins, many individuals survive these interactions but are often left with non-fatal, though potentially severe, wounds or scars. It has been reported that the interactions between dolphins and fisheries are probably the greatest conservation concern for dolphins [17]. In conclusion, skin mark prevalence may be an indicator of environmental or anthropogenic stressors in the ecosystem that may lead to individual and/or population-level health concerns [9,10].

In a recent study documenting skin conditions of the population of *S. c. taiwanensis* from 2006 to 2010, 37% of individuals showed evidence of fungal disease, various lesions, ulcers, and nodules [18]. Their results suggested the greater prevalence of compromised skin disorders may be associated with high levels of environmental pollution [18]. Despite the need for further monitoring of toxins in *S. c. taiwanensis*, the literature favors extensive habitat pollution leading to the accumulation of biological toxic substances within individuals, which diminishes the marine mammals’ capacity to reproduce as well as their immune response, thereby posing a serious threat to the health and viability of the species. A previous study [19] on *S. c. taiwanensis,* conducted from 2007 to 2010, showed that more than 30% of this population exhibited injuries caused by fishing gear. Another study [20] determined that more than half of the total observed individuals (n = 78, 2007–2015) examined in their research sustained significant injuries during human activities, with a total of 93 major injuries recorded on 46 individuals. This signifies that the potential risk of dolphin injuries inflicted by human activity is ongoing. It was supposed that *S. c. taiwanensis* likely faces imminent extinction if radical action is not taken to counter the threats posed by the local fisheries (particularly net fisheries) and other hazards identified for the subspecies [20].

In this study, with the aid of photo-ID, skin marks were visually assessed and quantified for *S. c. taiwanensis* off central Taiwan. The coastal waters of central Taiwan are one of most important habitats for this population, but there is an increasing amount of human activity in this area (reviewed in [5]). The objectives of our work were to (1) use photo-ID to classify and quantify the types of skin marks observed on *S. c. taiwanensis* off central Taiwan from 2018 to 2019 and in 2021 using descriptions from previous studies and (2) estimate the overall skin mark prevalence among *S. c. taiwanensis* off central Taiwan and the risks they encounter. Mark presence may provide insight into the condition of individual humpback dolphins. Thus, deriving updated baseline estimates for the prevalence of the skin marks can be useful for tracking population health.

## 2. Materials and Methods

**Figure 1:**
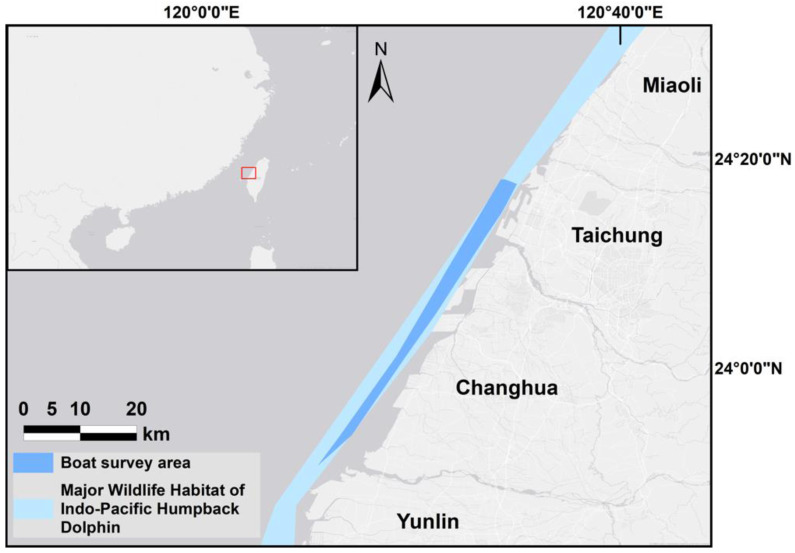
The major wildlife habitat of the Indo-Pacific humpback dolphin and the boat survey area along the west coast of central Taiwan.

In 2020, the Ocean Affairs Council in Taiwan announced a “Major Wildlife Habitat of Indo-Pacific Humpback Dolphin” as an area within 1–3 nautical miles offshore from the west coast of Taiwan, from 24°42′ N to 23°26′ N. The major wildlife habitat involves four counties: Miaoli, Taichung, Changhua, and Yunlin. This study focused on two counties located within the central habitat area: Taichung County (24°58′ N~24°11′ N) and Changhua County (24°11′ N~23°51′ N) (Figure 1). The study area included waters from the shoreline to shallow waters with depths < 15 m. Boat-based photo-ID surveys were conducted in 2018 (May to September), 2019 (April to September), and 2021 (April to November) by traveling at an average speed of 6 to 9 knots (13.89 km/h), with an average of 2 to 3 knots (4.63 km/h) when approaching animals using 10~12-meter-long CT-1 fishing boats. Dolphins were photographed using digital single-lens reflex cameras (Canon, Olympus, Pentax, or Nikon with 70–300 mm zoom lenses or 400 mm prime lenses). Every image was cataloged and cut into shape using PhotoImpact 11software, and individuals were further identified from those images by their distinctive scars and markings. 

**Figure 2:**
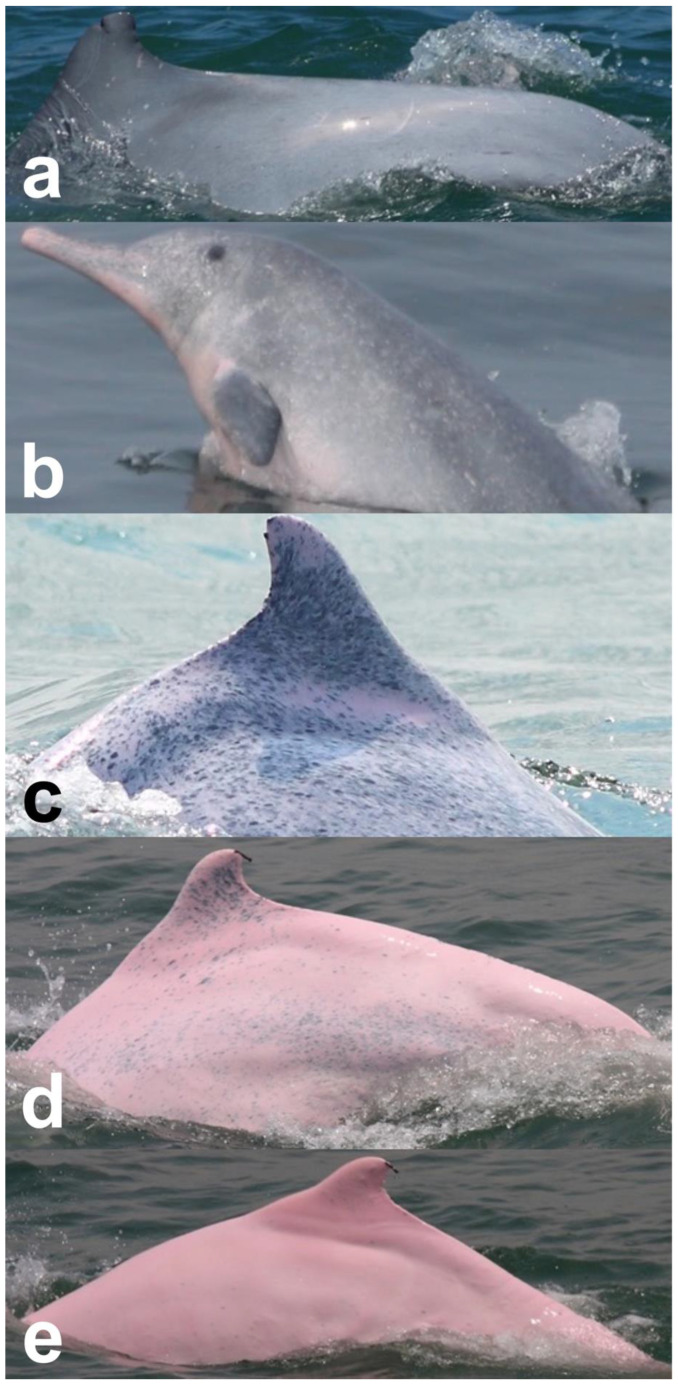
Images of *Sousa chinensis* in different coloration stages: (**a**) calf, (**b**) mottled stage, (**c**) speckled stage, (**d**) spotted adult, and (**e**) unspotted adult.

Identified individuals were grouped into five coloration stages based on changes in body color [21]: gray and unspotted (calf), gray with some white spotting (mottled), white with lots of black spotting (speckled), white with less black spotting (spotted adult), and white with a pinkish tint and no spotting (unspotted adult) (Figure 2). In this study, markings that possibly resulted from conspecifics or sharks were excluded from the analysis. Injuries were classified into five categories: dorsal fin/fluke mutilation, narrow-spaced linear marks, wide-spaced linear marks, back indentation, and others (Table 1). Skin lesions were classified into seven categories: nodules, hypertrophic scars, barnacles, pale, black patch, red patch, and orange/yellow patch (Table 2).

## 3. Results

In the three years (2018–2019 and 2021), 87 boat surveys were performed (43, 20, and 24 surveys, respectively), and 73,073 good-quality images were analyzed. In total, 50 individuals were identified in 2018, 28 individuals were identified in 2019, and 34 individuals were identified in 2021. In total, 57 distinctive individuals were identified. Among all 57 distinctive individuals, 17.5% (n = 10) were identified as calves, 29.8% (n = 17) were identified in the mottled stage, 28% (n = 16) were identified in the speckled stage, 21% (n = 12) were identified as spotted adults, and 3.5% (n = 2) were identified as unspotted adults. Spotted adults and unspotted adults were pooled into the adult stage for further analysis. Among the 57 distinctive individuals, 17 were resighted in all three years, including four mottled, nine speckled, and four spotted adults. Images of these 17 individuals were further analyzed for an interannual comparison.

### 3.1. Prevalence of Injuries

**Figure 3:**
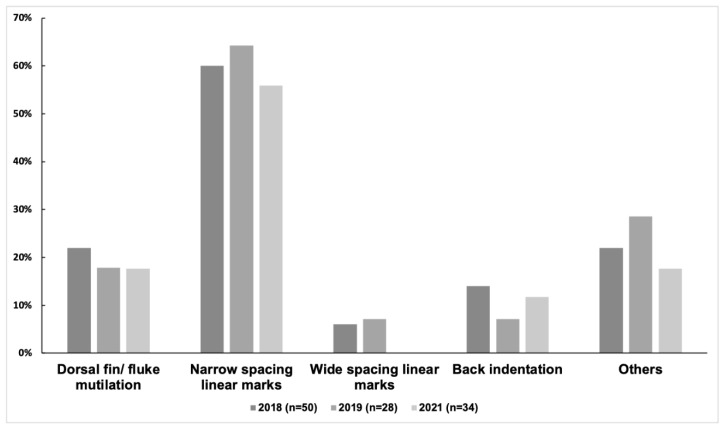
The prevalence of different injury categories in each year (n = 50, 28, and 34).

**Figure 4:**
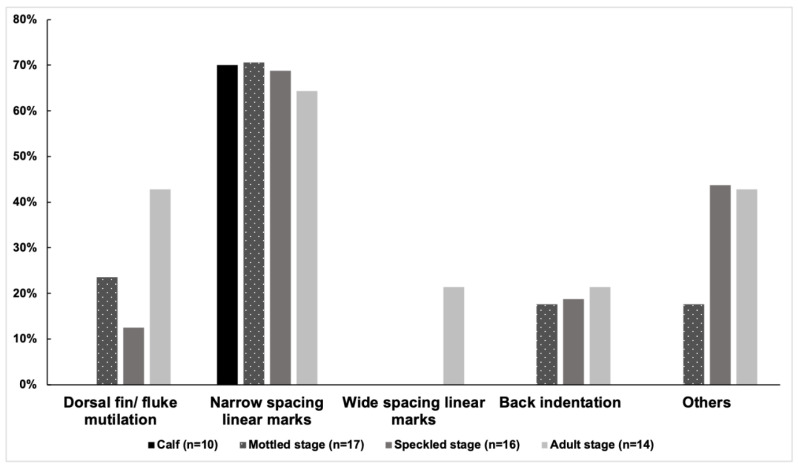
The prevalence of different injury categories in each coloration stage from 2018 to 2021 (n = 57).

At least one category of injury was observed in 47 of 57 distinctive individuals (82%) from 2018 to 2021. The prevalence of injuries was 80% (40/50) in 2018, 82% (23/28) in 2019, and 71% (24/34) in 2021. Among the 57 distinctive individuals, the prevalence of the five categories of injuries was as follows: narrow-spaced linear marks (68%), others (28%), dorsal fin/fluke mutilation (21%), back indentation (16%), and wide-spaced linear marks (5%). Narrow linear marks were the most prevalent injury in each year (Figure 3 and Table 3). Wide-spaced linear marks were not observed in 2021. The prevalence of injuries of the 57 distinctive individuals in each coloration stage was as follows: calf (70%, 7/10), mottled stage (76%, 13/17), speckled stage (81%, 13/16), and adult stage (100%, 14/14). The adult stage showed a higher prevalence of dorsal fin/fluke mutilation, wide-spaced linear marks, and back indentation (Figure 4). No new deep injuries were found in the 17 resighted individuals from 2018 to 2021, although one of them was observed with a new linear wound on its upper jaw in 2019.

### 3.2. Prevalence of Skin Lesions

**Figure 5:**
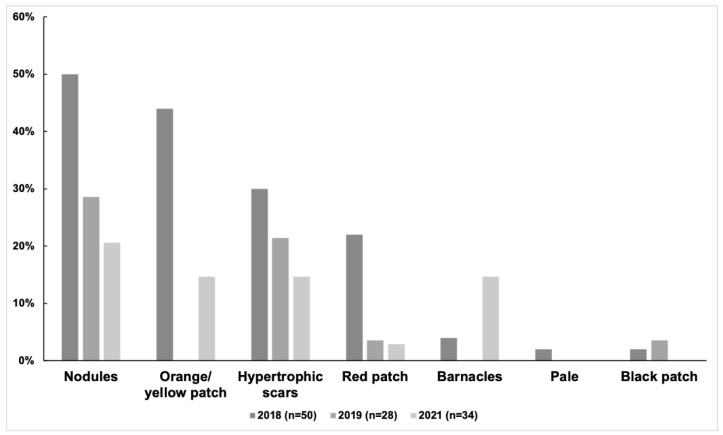
The prevalence of different skin lesion categories in each year (n = 50, 28, and 34).

**Figure 6:**
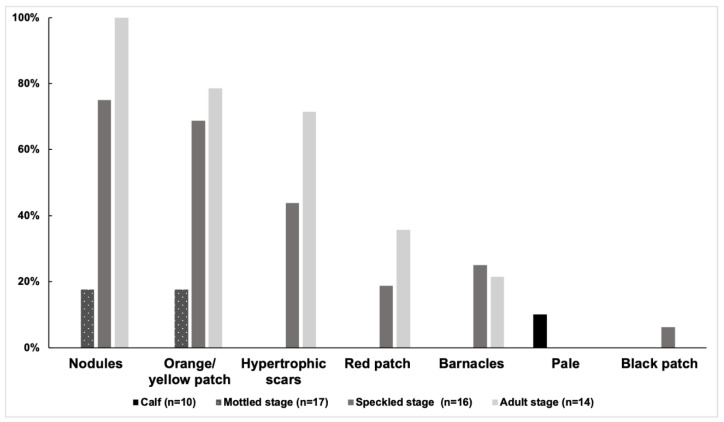
The prevalence of different skin lesion categories in each coloration stage from 2018 to 2021 (n = 57).

**Figure 7:**
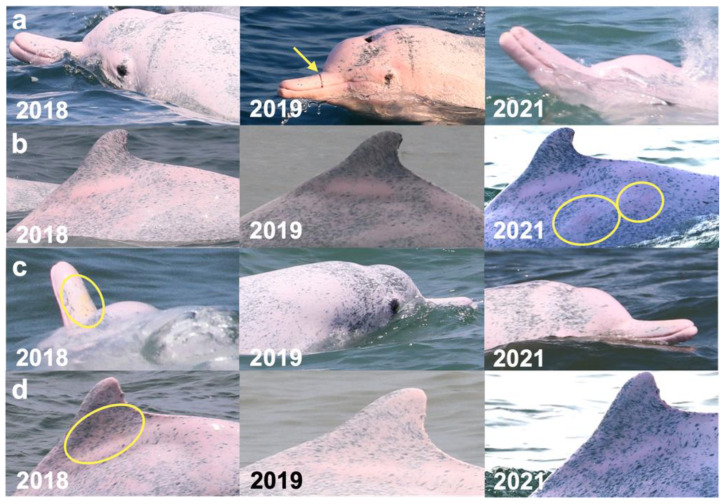
Images of 4 of the 17 resighted individuals in 2018, 2019, and 2021. (**a**) A speckled individual was observed with a new linear wound on its upper jaw only in 2019. (**b**) A speckled individual was found with a new red patch on its left side only in 2021. (**c**) A spotted adult was observed with a yellow patch on its upper jaw only in 2018. (**d**) A speckled-stage individual was found with a yellow patch on the left side of its dorsal fin only in 2018. Arrows and circles: injury or lesion.

At least one category of skin lesion was observed in 33 of 57 distinctive individuals (58%). The prevalence of skin lesions was 62% (31/50) in 2018, 39% (11/28) in 2019, and 41% (14/34) in 2021. Among the 57 distinctive individuals, the prevalence of the six categories of skin lesions was as follows: nodules (51%), orange/yellow patch (44%), hypertrophic scars (30%), barnacles (12%), white patch (2%), and black patch (2%). Nodules were the most prevalent skin lesion in each year (Figure 5 and Table 4). A pale lesion was observed in only one individual in three years, and a black patch only appeared in another individual sighted in both 2018 and 2019. Orange/yellow patches and barnacles were not observed in 2019. The prevalence of skin lesions of the 57 distinctive individuals in each age class was as follows: calf (10%, 1/10), mottled (24%, 4/17), speckled (88%, 14/16), and adult stage (100%, 14/14). The adult stage showed a higher prevalence of nodules, orange/yellow patches, hypertrophic scars, and red patches (Figure 6). Among the 17 resighted individuals, no skin lesions were noticed in three individuals. In addition, at least one category of skin lesion was found in seven individuals in all three years, while other individuals showed different conditions of lesion remission and relapse (Figure 7). 

## 4. Discussion

There was no consistency in the age reported for dolphins among previous studies on skin scars, and age-class documentation from photo-ID data was usually limited to adult or calf distinctions, which are of limited use in terms of information for ailments affecting sub-adults [11]. Calves of *S. chinensis* are dark gray or black at birth, and their coloration lightens through a mottled and speckled gray as they age, becoming juveniles and sub-adults, while adults are spotted and unspotted light gray to white [21]. Since most species of dolphins do not dramatically change coloration with age, the presumed age-class information in *S. chinensis* provides a good chance to understand the epidemiological factors influencing the prevalence of skin marks and different mark types in coastal dolphins. 

*S. chinensis*, similar to certain coastal cetacean species, is predominantly a predator and is hence subjected to indirect hazards such as fisheries bycatch, living space destruction due to land reclamation and pollution, reduced water discharge into sea-like water diversions, and noise pollution underwater [2]. These factors contribute to the skin lesions of *S. chinensis*. Differences in the salinity and temperature of the water are assumed to be important factors in the skin lesions in *S. chinensis*. However, this could not explain why there is a difference in the prevalence of skin conditions among the different coloration stages. Among other dolphins, similar skin conditions have been supposed to be associated with abnormally high levels of residual contaminants in fat tissues [12]. Compromised immune function associated with environmental pollutants has been considered [30,31]. Documented evidence stated that cetaceans in the Taiwanese region have comparatively lower levels of polychlorinated biphenyls (PCBs), mercury (Hg), and polybrominated diphenyl ethers (PBDEs) [32,33,34], but later it was proven that these water bodies are contaminated with heavy metals such as silver (Ag) and Cadmium (Cd), which are potential health threats [35,36,37]. The higher prevalence of skin lesions in this study provides important supporting evidence for the bioaccumulation of pollutants. A greater prevalence of skin lesions was found in mature animals, and a moderate-to-low prevalence of skin lesions was found among immature animals. Similar findings were reported in a previous study conducted between 2006 and 2010 on the whole population [18]. Furthermore, the prevalence of skin lesions in this study was higher than that in the previous study (from calf to unspotted: 7.7%, 32%, 37.9%, 92.3%, and 75% in [18] vs. 10%, 24%, 88%, 100%, and 100% in this study), indicating potentially significant health risks in this population. The prevalence is comparable to the previous studies on coastal cetacean populations across the globe (reviewed in [18]). It is asserted that there is a need for monitoring programs for pollutant concentrations in *S. c. taiwanensis* and its habitats, which is imperative to preserve the species and the coastal ecosystem. In addition, the monitoring of pathogens and their epidemiological patterns associated with stress and immunosuppression in dolphins serves as an indicator of ocean health [38]. As the total population comprises dozens of individuals, radical measures to conserve the population and reduce environmental hazards are considered indispensable.

In this study, nodules were highly prevalent (51%) and presented as circumscribed and swollen skin lumps. Orange films were the next most commonly prevalent (44%) lesion in this study. The prevalences of nodules and orange films in the previous study [18] were 15.5% and 11.3%, respectively. The known potentially etiological agents of skin nodules in odontocetes include fungi (*Lacazia loboi*, *Fusarium* spp., *Paracoccidioides brasiliensis,* and *Trichophyton* spp.), the bacteria *Streptococcus iniae*, and papillomaviruses [39]. Bottlenose dolphins (*Tursiops truncatus*) infected by *L. loboi* from the Indian River Lagoon, Florida, demonstrated marked impairment in adaptive immunity that can be potentially related to prolonged exposure to these environmental stressors. On the other hand, the salinity and temperature fluctuations of water also play their role in susceptibility to infection [12]. Further studies are necessary to investigate the etiology and to assess the impact of cutaneous nodules on the long-term health of affected *S. c. taiwanensis*. The orange films were a direct manifestation of a diatom, which is true in the case of other cetacean species [40]. Diatom attachment, by itself, is not a skin ailment that is an infectious condition [11], but due to the accumulation of diatoms there is slower skin regeneration following any insult and there is also a compromised swimming speed that negatively influences the physical condition and performance of the affected animal. This is an important factor in the decline of this critically endangered population. Pale lesions are either circular or amorphous, with rounded edges and a white-to-matte appearance, which indicates viral infection or inflammation. A black patch is similar in appearance to the poxvirus infection lesions described in previous studies [13,15]. Whether pale or black patches are significant to dolphin health, the current study showed that these two lesions were found much less (one individual for each) compared to the previous study conducted between 2006 and 2010 (10 and 3 individuals, respectively) [18]. 

A narrow linear mark might barely pose a threat to a dolphins’ health and survival, although it still indicates dolphin–fishery interactions. In contrast, the other marks, such as mutilation, wide-spaced linear marks, and V-shaped indentations, have been associated with deep injuries attributed to human interactions and with different degrees of severity [24]. When excluding narrow-spaced linear marks, the prevalence of the severe injuries was 38% (19/50) in 2018, 39% (11/28) in 2019, and 38% (13/34) in 2021, and the prevalence of injuries of the 57 distinctive individuals in each coloration stage was calf (0%, 0/10), mottled stage (47%, 8/17), speckled stage (56%, 9/16), and adult stage (79%, 11/14). The prevalence in mottled, speckled, and adult individuals was comparable to the previous study on this population [20] and higher than other previous reports for small cetaceans [24,41,42]. Dolphins exhibit an extraordinary capacity to heal deep soft-tissue injuries such as shark bites and anthropogenic trauma, and the deep wounds in the dolphins may heal in a regenerative manner rather than repair [43]. However, these scars significantly alter the physiology and behavior of the dolphins and occasionally lead to death [42]. For example, a juvenile male *S. c. taiwanensis* was found stranded in south Taiwan on 21 January 2022, with injuries caused by past gillnet entanglement. The necropsy report showed that the acute bacterial infection and severe blunt force trauma were the direct causes of death, while the past gillnet entanglement injuries may be the contributory cause (WCY, personal communication). Furthermore, dolphins caught in driftnets may sustain injuries inflicted by fishermen, such as deep indentations or mutilations of stuck appendices (reviewed in [10]). This results in serious infections that may reduce swimming activities, causing higher energy expenditure and starvation and leading to death. Previous studies on *T. truncatus* and other species demonstrated a relationship between the incidence of injury and fishery activities and that the occurrence of skin injuries proportionally increased with higher fishery interactions [24,42]. The prevalence of deep injuries, which can be expected to reduce health and survivorship, in the current study was similar or lower than those in previous studies [20,44], although there were some differences in the survey effort, survey area, and injury classification method. In addition, no new deep injuries were found in the 17 individuals from 2018 to 2021. However, there are potential under-representations of the impacts since fatal injuries could not be observed in this study. For example, the severe injury classified as wide-spaced linear marks, which are likely the result of a turning propeller hit or net entanglement, were not observed in 2021. Furthermore, the preliminary investigation (data not shown) indicated that this population was at risk of predation from sharks, and it warrants further study on the correlation between anthropogenic injuries and shark bites. Our results can serve as a baseline for future analyses of injuries among the whole population. Further research should document the statistics of fatal injuries among stranded individuals and determine to what level the injuries can influence the health and survival of this critically endangered population.

## 5. Conclusions

It has been reported that the cetaceans habituating the Taiwanese waters may be affected by immunosuppression resulting from multiple anthropogenic stress factors, including fishery bycatch, the vessel movement rate, whale-watching activities, water and vessel noise, and land-based pollution [45]. The moderate-to-high prevalence of skin marks in this study is designated as a warning of the risks. Our results offer important recommendations, and further studies are warranted to examine if the situation is also found in the whole population of *S. c. taiwanensis*. Apart from our current efforts, consistently collecting photos and photo-ID data from free-ranging cetaceans would be highly recommended for other countries along the western Pacific region. Further studies investigating the skin marks of free-ranging cetaceans will help us to understand the crucial and potential threats to cetaceans, the ocean, and human health.

## Figures and Tables

**Table 1:** Definitions of injury (arrows and circles) categories and potential causes.

Injury Categories	Description	Examples	Potential Causes	References
Dorsal fin/fluke mutilation	Missing the apex of the dorsal fin/fluke, with a sharp edge or a linear cut down the dorsal fin	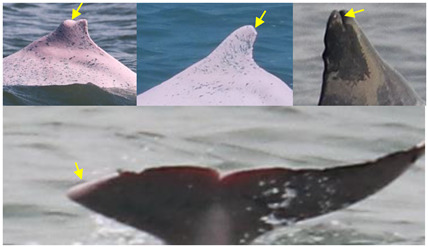	cut from net entanglement or other object	[10,22,23]
Narrow-spaced linear marks	Serial linear marks, including both shallow and deep marks, especially those appearing along the spine of the peduncle	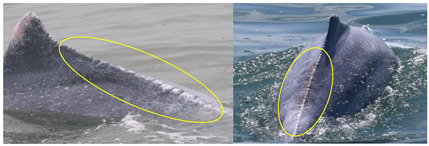	net entanglement or inherent deformities	[23,24]
Wide-spaced linear marks	Serial linear marks that extend from one side of the peduncle to the other side, with a long and equal interval between each mark	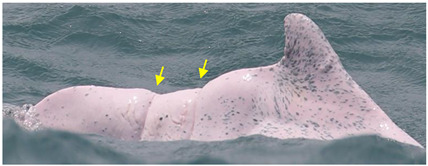	net entanglement or ship strike	[23,24]
Back indentation	V-shaped indentation located along the spine; size varies	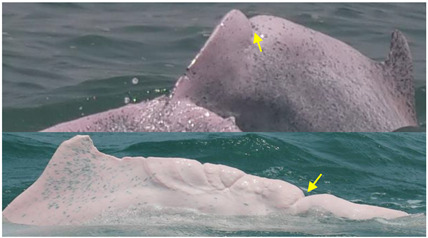	net entanglement or ship strike	[10,23,25]
Others	Marks seemingly unrelated to natural causes and that cannot be assigned to the other scar categories	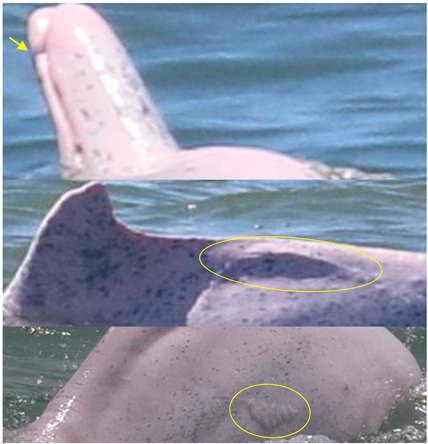	cut from net entanglement or other object	[26]

**Table 2:** Definitions of skin lesion (arrows and circles) categories and potential causes.

Skin Lesion Categories	Description	Examples	Potential Causes	References
Nodules	Oval or circular cutaneous elevations of the skin	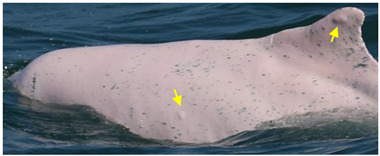	fungal or bacterial infection	[27]
Hypertrophic Scars	Raised lesions with oval shapes	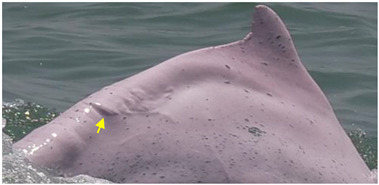	infection or immune deficiency	[22]
Barnacles	One or multiple hanging barnacles, especially seen on dorsal fins and flukes	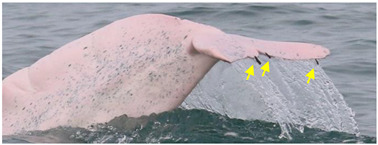	swimming slowly due to health issues	[28]
Pale	White in color and irregular in shape; diffuse edges and of any size	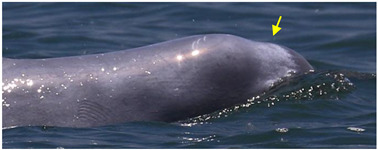	fungal infection	[9,22]
Black patch	Uniform in color and irregular in shape; diffuse edges	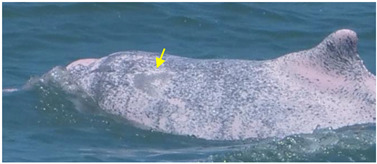	pox virus infection	[9,22,25]
Red patch	Pink patch with multiple small red spots scattered around; irregular in shape	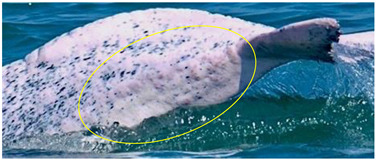	bacterial or viral infection	[27]
Orange/yellow patch	Orange or yellow coloration with diffuse edges	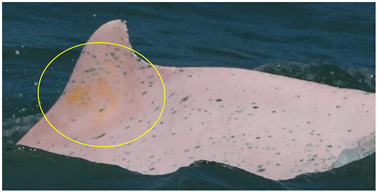	attached diatom	[22,27,29]

**Table 3:** Prevalence of injuries in 2018, 2019, and 2021.

Stage (n = 2018, 2019, 2021)	Dorsal Fin/Fluke Mutilation	Narrow-Spaced Linear Marks	Wide-Spaced Linear Marks	Back Indentation	Others
Calf (n = 5,3,8)	(0,0,0)	(3,2,4)	(0,0,0)	(0,0,0)	(0,0,0)
Mottled stage (n = 16,7,11)	(3,1,2)	(10,5,5)	(0,0,0)	(2,0,1)	(1,2,1)
Speckled stage (n = 15,10,10)	(2,2,2)	(9,7,6)	(0,0,0)	(3,2,2)	(4,4,3)
Spotted adult (n = 12,7,5)	(6,2,2)	(7,4,4)	(2,2,0)	(2,0,1)	(5,2,2)
Unspotted adult (n = 2,1,0)	(0,0,0)	(1,0,0)	(1,0,0)	(0,0,0)	(1,0,0)

**Table 4:** Prevalence of skin lesions in 2018, 2019, and 2021.

Stage (n = 2018, 2019, 2021)	Nodules	Orange/Yellow Patch	Hypertrophic Scars	Red Patch	Barnacles	Pale	Black Patch
Calf (n = 5,3,8)	(0,0,0)	(0,0,0)	(0,0,0)	(0,0,0)	(0,0,0)	(1,0,0)	(0,0,0)
Mottled stage (n = 16,7,11)	(1,1,1)	(2,0,1)	(0,0,0)	(0,0,0)	(0,0,0)	(0,0,0)	(0,0,0)
Speckled stage (n = 15,10,10)	(11,4,2)	(9,0,3)	(5,4,3)	(6,0,1)	(0,0,4)	(0,0,0)	(1,1,0)
Spotted adult (n = 12,7,5)	(11,3,4)	(9,0,1)	(9,2,2)	(5,1,0)	(1,0,1)	(0,0,0)	(0,0,0)
Unspotted adult (n = 2,1,0)	(2,0,0)	(2,0,0)	(1,0,0)	(0,0,0)	(1,0,0)	(0,0,0)	(0,0,0)

## Data Availability

Not applicable.
